# Phenotypic features of genetically modified *DMD*-X^KO^X^WT^ pigs

**DOI:** 10.1016/j.reth.2023.09.010

**Published:** 2023-09-20

**Authors:** Kazutoshi Okamoto, Hitomi Matsunari, Kazuaki Nakano, Kazuhiro Umeyama, Koki Hasegawa, Ayuko Uchikura, Shuko Takayanagi, Masahito Watanabe, Jun Ohgane, Michael Stirm, Mayuko Kurome, Nikolai Klymiuk, Masaki Nagaya, Eckhard Wolf, Hiroshi Nagashima

**Affiliations:** a Laboratory of Medical Bioengineering, Department of Life Sciences, School of Agriculture, Meiji University, 1-1-1 Higashimita, Tama-ku, Kawasaki, Kanagawa 214-8571, Japan; b Meiji University International Institute for Bio-Resource Research, 1-1-1 Higashimita, Tama-ku, Kawasaki, Kanagawa 214-8571, Japan; c Laboratory of Genomic Function Engineering, Department of Life Sciences, School of Agriculture, Meiji University, 1-1-1 Higashimita, Tama-ku, Kawasaki, Kanagawa 214-8571, Japan; d Chair for Molecular Animal Breeding and Biotechnology, Gene Center and Department of Veterinary Sciences, LMU Munich, 81377 Munich, Germany; e Center for Innovative Medical Models (CiMM), LMU Munich, 85764 Oberschleissheim, Germany

**Keywords:** Dystrophinopathy, Carrier, Duchenne muscular dystrophy, Hereditary disease, Large animal model, Pig

## Abstract

**Introduction:**

Duchenne muscular dystrophy (DMD) is a hereditary neuromuscular disorder caused by mutation in the dystrophin gene (*DMD*) on the X chromosome. Female DMD carriers occasionally exhibit symptoms such as muscle weakness and heart failure. Here, we investigated the characteristics and representativeness of female DMD carrier (*DMD-*X^KO^X^WT^) pigs as a suitable disease model.

**Methods:**

In vitro fertilization using sperm from a *DMD*-X^KO^Y↔X^WT^X^WT^ chimeric boar yielded *DMD-*X^KO^X^WT^ females, which were used to generate F2 and F3 progeny, including *DMD-*X^KO^X^WT^ females. F1–F3 piglets were genotyped and subjected to biochemical analysis for blood creatine kinase (CK), aspartate aminotransferase, and lactate dehydrogenase. Skeletal muscle and myocardial tissue were analyzed for the expression of dystrophin and utrophin, as well as for lymphocyte and macrophage infiltration.

**Results:**

*DMD*-X^KO^X^WT^ pigs exhibited various characteristics common to human DMD carrier patients, namely, asymptomatic hyperCKemia, dystrophin expression patterns in the skeletal and cardiac muscles, histopathological features of skeletal muscle degeneration, myocardial lesions in adulthood, and sporadic death. Pathological abnormalities observed in the skeletal muscles in *DMD*-X^KO^X^WT^ pigs point to a frequent incidence of pathological abnormalities in the musculoskeletal tissues of latent DMD carriers. Our findings suggest a higher risk of myocardial abnormalities in DMD carrier women than previously believed.

**Conclusions:**

We demonstrated that *DMD-*X^KO^X^WT^ pigs could serve as a suitable large animal model for understanding the pathogenic mechanism in DMD carriers and developing therapies for female DMD carriers.

## Highlights

• DMD carrier model pigs were produced using artificial reproductive technologies.
• *DMD*-X^KO^X^WT^ pigs share various characteristics with DMD carrier patients.
• Myocardial fibrosis in *DMD*-X^KO^X^WT^ pigs may underlie cardiac incompetency in carriers.

## 1 Introduction

Duchenne muscular dystrophy (DMD) is the most prevalent neuromuscular disorder, affecting up to 1/3600 male births worldwide [1]. Major symptoms of DMD include progressive muscle weakness and wasting [2]. Deficiency of dystrophin caused by mutation in the dystrophin gene (*DMD*) on the X-chromosome results in degeneration and necrosis of muscle fibers due to loss of integrity of the sarcolemma, thereby leading to respiratory insufficiency [3], heart failure [4], and premature death in the second to fourth decade of life [5].

Although females possessing heterozygous *DMD* mutations (DMD carriers) rarely exhibit clinical symptoms [6], they can sporadically develop muscle weakness and heart failure [[6], [7], [8]]. The incidence of cardiomyopathy in DMD carriers increases with age [9,10]. Adult DMD carriers therefore have been recommended to undergo echocardiography every 5 years [11,12]. The unique characteristics of carriers, including onset and sites of pathology, and severity of the symptoms, have yet to be investigated. However, studies involving DMD carriers can be hindered due to biased selection of subjects and the intentional avoidance of invasive examinations such as myocardial biopsy.

Animal disease models manifesting symptoms extrapolatable to human patients are indispensable for understanding pathogenic mechanisms and developing therapies [13,14]. Novel disease models in pigs have been intensively developed in recent studies due to pigs' advantages as an experimental animal with physiological and anatomical similarity to humans [15,16]. We have demonstrated that *DMD*-knockout (KO) pigs that faithfully manifest DMD symptoms [[17], [18], [19], [20]] can serve as a large animal model for translational studies, including gene therapy [21]. Thus, DMD carrier pigs may also provide clinically relevant insights.

We have developed a stable procedure for reproducing DMD carrier pigs through the use of cryopreserved boar sperm carrying the *DMD* mutation on the X-chromosome [22]. In the present study, we performed physiological and pathological analyses of female DMD carrier pigs to evaluate their characteristics and ability to model female patients.

## 2 Materials and methods

### 2.1 Experimental animals

All animal experiments were approved by the Institutional Animal Care and Use Committee of Meiji University (approval numbers: MUIACUC2020-111 and MUIACUC2020-125). All recombinant DNA experiments performed in this study were approved by the Genetic Modification Safety Committee of Meiji University (2018-6). All experiments were performed in accordance with institutional guidelines and regulations. Pigs were kept in a temperature-controlled room, fed food appropriate for their growth stage, and given free access to water.

### 2.2 Generation of *DMD*-X^KO^X^WT^ female pigs

We previously generated a male *DMD*-X^KO^Y↔X^WT^X^WT^ chimeric pig by blastocyst complementation using *DMD*-X^KO^Y male cells and wild-type (WT) female embryos [22]. This chimeric male pig produced fertile gametes: *DMD*-X^KO^ sperm and normal Y sperm [22]. To generate *DMD-*X^KO^X^WT^ female pigs, cryopreserved epididymal sperm collected from the *DMD-*X^KO^Y↔X^WT^X^WT^ chimeric boar were subjected to in vitro fertilization (IVF) as previously described [23] with slight modifications. Briefly, sperm (5 × 10^5^ cells/mL) and in vitro matured oocytes were cocultured in porcine fertilization medium (PFM; Research Institute for Functional Peptides, Yamagata, Japan) at 38.5 °C for 8 h under the following conditions: 5% CO_2_, 5% O_2_, 90% N_2_, and saturated humidity. Presumed zygotes were cultured using porcine zygote medium-5 (PZM-5, Research Institute for Functional Peptides) under the following conditions: 5% CO_2_, 5% O_2_, 90% N_2_, 38.5 °C, and saturated humidity. After the morula stage, 10% fetal bovine serum was added to the culture medium. Blastocyst-stage embryos cultured in vitro for 5–6 d after fertilization were surgically transferred into the uterine horns of estrus-synchronized female recipient pigs, which generated the F1 generation of *DMD*-X^KO^X^WT^ progeny. Natural mating or artificial insemination of the F1 *DMD*-X^KO^X^WT^ sows with wild-type boars, produced the F2 generation. *DMD*-X^KO^X^WT^ pigs from our previous study [22] were also used to produce the F2 generation. F2 *DMD*-X^KO^X^WT^ pigs were crossed with wild-type boars to produce the F3 generation.

### 2.3 Genotyping

Genomic DNA was extracted from the tail tissue of the F1–F3 piglets using a DNeasy Blood and Tissue Kit (QIAGEN, Hilden, Germany). PCR analysis was performed using standard methods. The primers 5′-GTCTTTCAGCCACTGATTGT-3′ and 5′-TTTATGAGTATTGAATTTCCATCCC-3′ were used for the detection of *DMD* exon 52, which is deleted *on the mutated X chromosome*. The PCR product was subjected to agarose gel electrophoresis to confirm whether the test individuals were WT, *DMD-*X^KO^X^WT^, or *DMD-*X^KO^Y.

### 2.4 Blood biochemical analysis

Blood samples were collected from the ear vein and allowed to clot before the serum was separated by standard methods. Creatine kinase (CK), aspartate aminotransferase (AST), and lactate dehydrogenase (LDH) were measured using a dry-chemistry analyzer (FUJI DRI-CHEM 7000, FUJIFILM, Tokyo, Japan).

### 2.5 Tissue analysis

The biceps femoris and the left ventricle of *DMD-*X^KO^X^WT^ female pigs were used in the analysis of skeletal muscle and myocardial tissue, respectively. Control tissues were obtained from WT and *DMD-*X^KO^Y littermates. The tissues were mounted on cork bases using tragacanth gum (FUJIFILM Wako Pure Chemical, Osaka, Japan) and frozen in isopentane cooled in liquid nitrogen. Frozen tissues were sliced at a thickness of 8 μm and stained.

We analyzed the expression of the following three proteins by immunofluorescence staining: (i) dystrophin, an essential cytoskeletal protein for the maintenance of skeletal muscle and myocardium [24]; (ii) utrophin (UTRN), a homolog of dystrophin whose expression is upregulated in DMD patients [25]; and (iii) laminin subunit alpha-2 (*LAMA2*), a component of the basal lamina of the extracellular matrix [26].

The primary antibodies used were monoclonal anti-human dystrophin (NCL-DYS2, 1:100 dilution, Leica Biosystems, Wetzlar, Germany), which has been validated for staining porcine samples [19]. For staining utrophin and laminin alpha-2, we used polyclonal anti-mouse utrophin (ABN1739, 1:100 dilution; Merck Millipore, Darmstadt, Germany) [27], and monoclonal anti-mouse laminin alpha-2 (sc-59854, 1:200 dilution; Santa Cruz Biotechnology, Dallas, TX, USA) [28]. To our knowledge, the cross-reactivity of these antibodies to porcine antigens has not been reported. According to the antibody manufacturer's instruction, however, an antibody validated for mouse will cross-react with pig, if the homology of the immunogen sequence between mouse and pig is higher than 85%. Based on BLAST search (https://blast.ncbi.nlm.nih.gov/Blast.cgi), porcine utrophin and laminin alpha-2 have 88.5% and 90.0% homology with mouse antigens, respectively. We therefore used the ABN1739 and sc-59854 antibodies in the present study.

Secondary antibodies labelled with Alexa Fluor family of fluorescent dyes (Thermo Fisher Scientific, Waltham, MA, USA) were diluted 1:300 and used in the following combinations: Alexa Fluor 488 (A11029) and Alexa Fluor 594 (A21207) for double staining of dystrophin and utrophin, Alexa Fluor 488 (A11029) and Alexa Fluor 647 (A21247) or double staining of dystrophin and laminin. After antibody treatment, sections were mounted in Vectashield mounting medium (H-1200; Vector Laboratories, Newark, CA, United States) containing 4′,6-diamidino-2-phenylindole (DAPI) for nuclear staining.

Immunohistochemical staining was performed to examine the presence of lymphocyte and macrophage infiltration in muscle tissue. As primary antibodies, anti-CD3 (IS503, Dako, Santa Clara, CA, USA) was used for the detection of T lymphocytes, and anti-Iba1 (019-19741, FUJIFILM Wako Pure Chemical) was used for the detection of macrophages. Primary antibodies were detected using horseradish peroxidase conjugated secondary antibodies (K4061, Dako). A liquid DAB + substrate chromogen system (K3468, Dako) was used as the chromogenic substrate. Hematoxylin–eosin (HE) staining was performed by standard procedures.

Stained sections were observed under a fluorescence microscope (BZ-X700; Keyence, Osaka, Japan).

### 2.6 Quantification of dystrophin expression levels in muscle tissue and the fibrotic area in the myocardium

Dystrophin expression in the skeletal muscle and myocardium of *DMD-*X^KO^X^WT^ female pigs was quantified from immunostaining images. Laminin, similar to dystrophin, is expressed in the sarcolemma. Normal muscle fibers of WT pigs are positive for both laminin and dystrophin staining. In *DMD-*X^KO^X^WT^ pigs, muscle fibers lacking dystrophin expression are positive for laminin and negative for dystrophin staining. Thus, differences in dystrophin expression patterns between *DMD-*X^KO^X^WT^ and WT pigs can be quantitatively compared by calculating the difference in the dystrophin/laminin fluorescence intensity ratio. We performed double staining of dystrophin and laminin, and 10 random fields were photographed at 20 × magnification using a fluorescence microscope (BZ-X700; Keyence). The luminance of dystrophin and laminin was measured using ImageJ software v1.53 (National Institutes of Health, Bethesda, MD, USA), the luminance ratio of dystrophin/laminin was determined, and the value relative to the ratio in WT samples was calculated.

Sirius Red staining was performed to detect myocardial fibrosis, and the fibrotic area was quantified using ImageJ software.

### 2.7 Statistical analysis

Statistical analysis was performed using SPSS software v26 (IBM SPSS, Chicago, IL, USA). Data are presented as the mean ± standard deviation (SD) or mean ± standard error of the mean (SEM). Welch's *t*-test was used to evaluate differences in numerical data between *DMD-*X^KO^X^WT^ and WT pigs. Differences were considered statistically significant at p < 0.05.

## 3 Results

### 3.1 Generation of *DMD-*X^KO^X^WT^ female pigs

We surgically transferred 22 blastocysts produced by in vitro fertilization using sperm from the *DMD*-X^KO^Y↔X^WT^X^WT^ chimeric boar to one female recipient, which resulted in five F1 offspring, including three *DMD-*X^KO^X^WT^ females and two WT males. All three F1 *DMD-*X^KO^X^WT^ females obtained reached sexual maturity. One of them was mated with a WT boar to produce F2 progeny. The other two were used for collecting samples of sexually mature individuals.

Furthermore, F1 *DMD-*X^KO^X^WT^ females from our previous study [22] were used to produce F2 progeny. Five F1 *DMD-*X^KO^X^WT^ females that had farrowed six times in total produced 60 F2 progeny, including 17 (28.3%) *DMD-*X^KO^X^WT^ individuals. One of the F2 *DMD-*X^KO^X^WT^ females was mated to a WT boar, resulting in the birth of seven more F3 progeny, of which two (28.6%) were *DMD-*X^KO^X^WT^ females. *DMD-*X^KO^X^WT^ pigs and WT littermates produced through the above process were used in this study.

### 3.2 Sudden onset in *DMD-*X^KO^X^WT^ female pigs

Although most human DMD carriers are asymptomatic, clinical symptoms can sporadically present at a wide range of ages [6]. Accordingly, the *DMD-*X^KO^X^WT^ pigs were reared for up to a maximum of 32 months, during which we monitored growth rate, CK level fluctuations, and DMD symptom development.

**Fig. 1:**
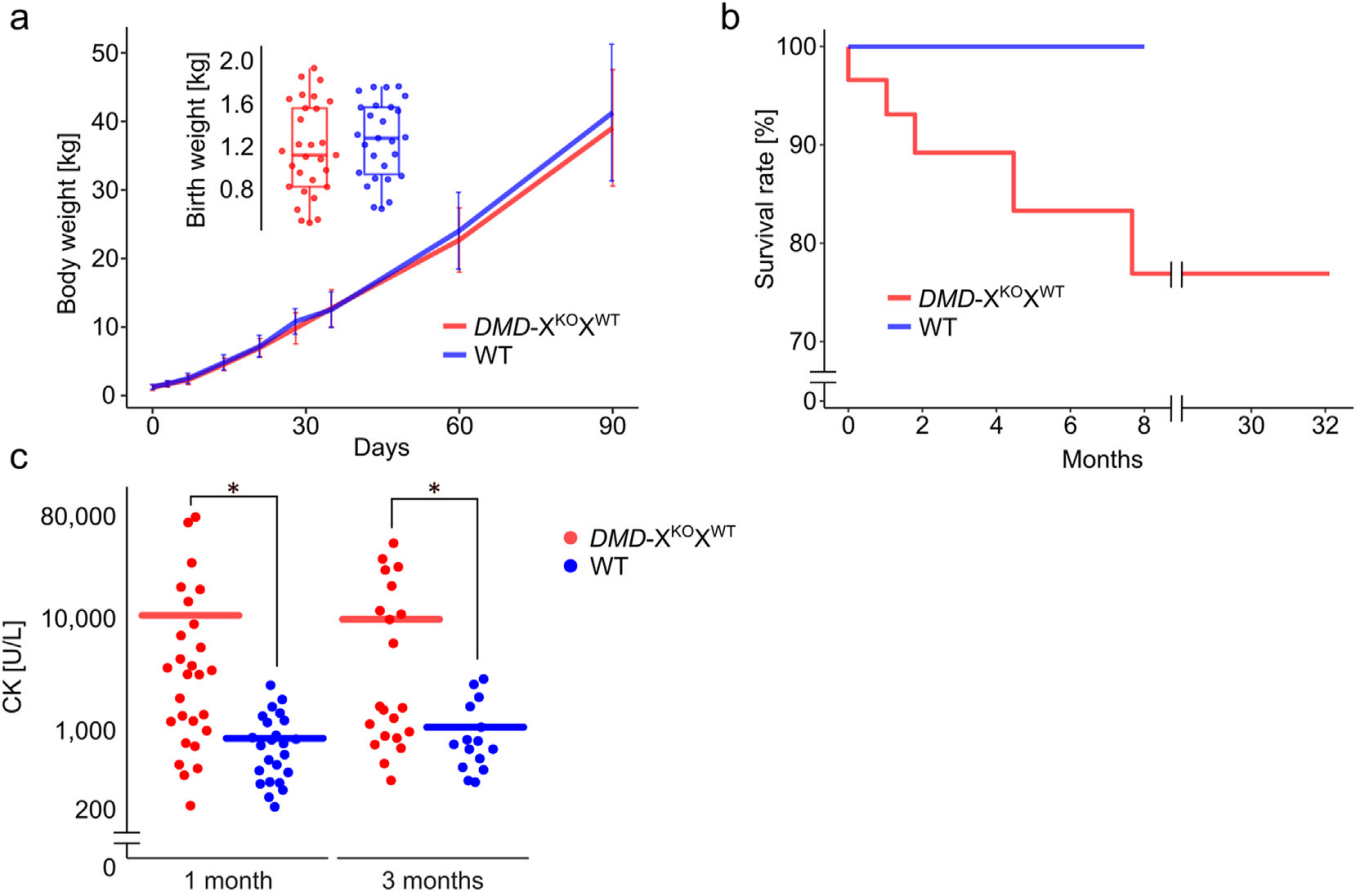
Growth and serum CK levels of *DMD-*X^KO^X^WT^ female pigs. a, b: Growth (a) and survival curve (b) of *DMD-*X^KO^X^WT^ pigs (red line, n = 29) compared to WT littermates (blue line, n = 27). c: Serum CK levels of young *DMD-*X^KO^X^WT^ female pigs. ∗p < 0.05 vs. WT individuals. Samples were collected from *DMD-*X^KO^X^WT^ pigs at 1 month (n = 27) and 3 months (n = 21) of age (red plots). WT pigs at 1 month (n = 24) and 3 months (n = 15) of age were used as the respective controls (blue plots). CK: creatine kinase. WT: wild type.

The birth weights of *DMD-*X^KO^X^WT^ pigs and WT littermates were 1.174 ± 0.08 kg (n = 29) and 1.254 ± 0.07 kg (n = 27), respectively, with no significant difference between the two groups. *DMD-*X^KO^X^WT^ pigs exhibited no differences in weight gain (Fig. 1a) or appearance compared to WT pigs.

Five (17.2%) of the 29 *DMD-*X^KO^X^WT^ pigs died during rearing (Fig. 1b). One died from prostration shortly after birth, and three died suddenly at 1, 2, and 4 months of age. Autopsies were not performed due to the time elapsed postmortem. The hind limbs of a 7-month-old *DMD-*X^KO^X^WT^ pig collapsed during mating with a WT male, and the female subsequently died. Autopsy of this individual revealed skeletal muscle degeneration and myocardial fibrosis (Supplementary Fig. S1). On the other hand, there were no deaths among WT littermates.

Multiple blood tests were performed on the 27 *DMD-*X^KO^X^WT^ pigs that survived at least 1 month after birth, and a wide range of serum CK levels (207–75,990 U/L) was observed among individuals of the same age (Supplementary Fig. S2a). The mean CK levels of young *DMD-*X^KO^X^WT^ pigs were significantly higher than those of age-matched WT littermates (1 month old: 10,167 ± 19,145 vs. 821 ± 561 U/L; 3 months old: 9376 ± 12,843 vs. 1031 ± 780 U/L, p < 0.05; Fig. 1c). Those with high CK levels tended to also have high AST and LDH levels (Supplementary Fig. S2b). The CK levels of *DMD-*X^KO^X^WT^ pigs tended to decrease with age (Supplementary Fig. S2a).

### 3.3 Histological analysis of the skeletal muscles of asymptomatic *DMD-*X^KO^X^WT^ female pigs

**Fig. 2:**
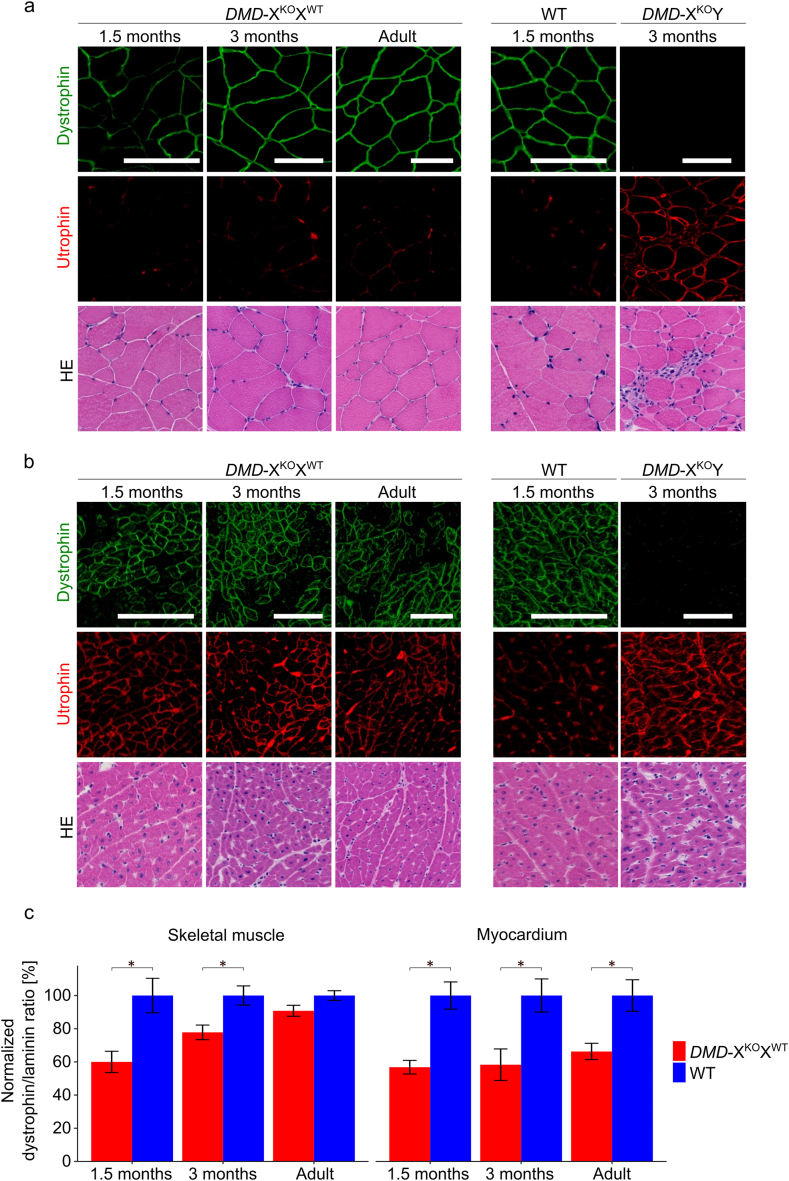
Histological analysis of the skeletal muscle and myocardium of *DMD-*X^KO^X^WT^ pigs. a, b: Immunostaining of dystrophin and utrophin in skeletal muscle (a) and myocardium (b) of *DMD-*X^KO^X^WT^ pigs. The upper and middle images of both panels exhibit fluorescent immunohistochemical staining of dystrophin and utrophin, respectively, and the lower images indicate HE staining. Scale bars = 100 μm. b: Partial upregulation of utrophin expression on the sarcolemma, a known myopathological feature of DMD. c: Dystrophin expression levels in *DMD-*X^KO^X^WT^ pigs (red bars). Dystrophin/laminin fluorescence intensity ratios were normalized to WT muscle tissues (blue bars). The graph represents the mean ± SEM. ∗p < 0.05 vs. WT pigs. Samples were collected from *DMD-*X^KO^X^WT^ pigs at 1.5, 3, and 14–32 months of age (n = 3, 3, and 6, respectively). WT pigs at 1.5, 3, and 6–8 months of age (n = 2, 3, 3, respectively) were used as the respective controls. HE: hematoxylin–eosin. WT: wild type.

Immunohistological and histopathological analyses of skeletal muscle tissue were performed in 12 asymptomatic *DMD-*X^KO^X^WT^ pigs at 1.5–32 months of age. The skeletal muscle tissue of 1.5-month-old *DMD-*X^KO^X^WT^ pigs (n = 3) exhibited a mosaic pattern of dystrophin staining. In contrast, the skeletal muscle tissue of 3-month-old individuals (n = 3) exhibited a uniform pattern of staining (Fig. 2a), which indicated that dystrophin was continuously distributed around the muscle fibers (sarcolemma). A similar pattern of dystrophin staining was observed in the skeletal muscle tissue of all older individuals (14–32 months old, n = 6; Fig. 2a). As in the WT animals, only a weak utrophin expression was detected in the skeletal muscle tissue of *DMD-*X^KO^X^WT^ pigs, regardless of age. At young ages (1.5–3 months old), the dystrophin expression levels in the skeletal muscle of *DMD-*X^KO^X^WT^ pigs were lower than those of WT individuals (p < 0.05, Fig. 2c, Supplementary Fig. S3) and tended to increase with age (Fig. 2c). Degeneration of muscle fibers was observed in 3 of the 6 young individuals (Supplementary Fig. S4a). On the other hand, the skeletal muscle tissue of adult pigs exhibited no degeneration, and infiltration of immune cells was observed only in 1 of 6 individuals (Supplementary Fig. S4b).

### 3.4 Histological assessment of the myocardium of asymptomatic *DMD-*X^KO^X^WT^ female pigs

Immunohistological and histopathological analyses of myocardial tissue were performed in 12 asymptomatic *DMD-*X^KO^X^WT^ pigs at 1.5–32 months of age. Dystrophin immunostaining in *DMD-*X^KO^X^WT^ pigs revealed a mosaic pattern, exhibiting a mixture of dystrophin-positive and dystrophin-negative cells (Fig. 2b). The ratio of dystrophin/laminin fluorescent intensities indicated that the dystrophin levels in the myocardium of *DMD-*X^KO^X^WT^ pigs at all ages were significantly lower than those of WT pigs (p < 0.05, Fig. 2c, Supplementary Fig. S3).

**Fig. 3:**
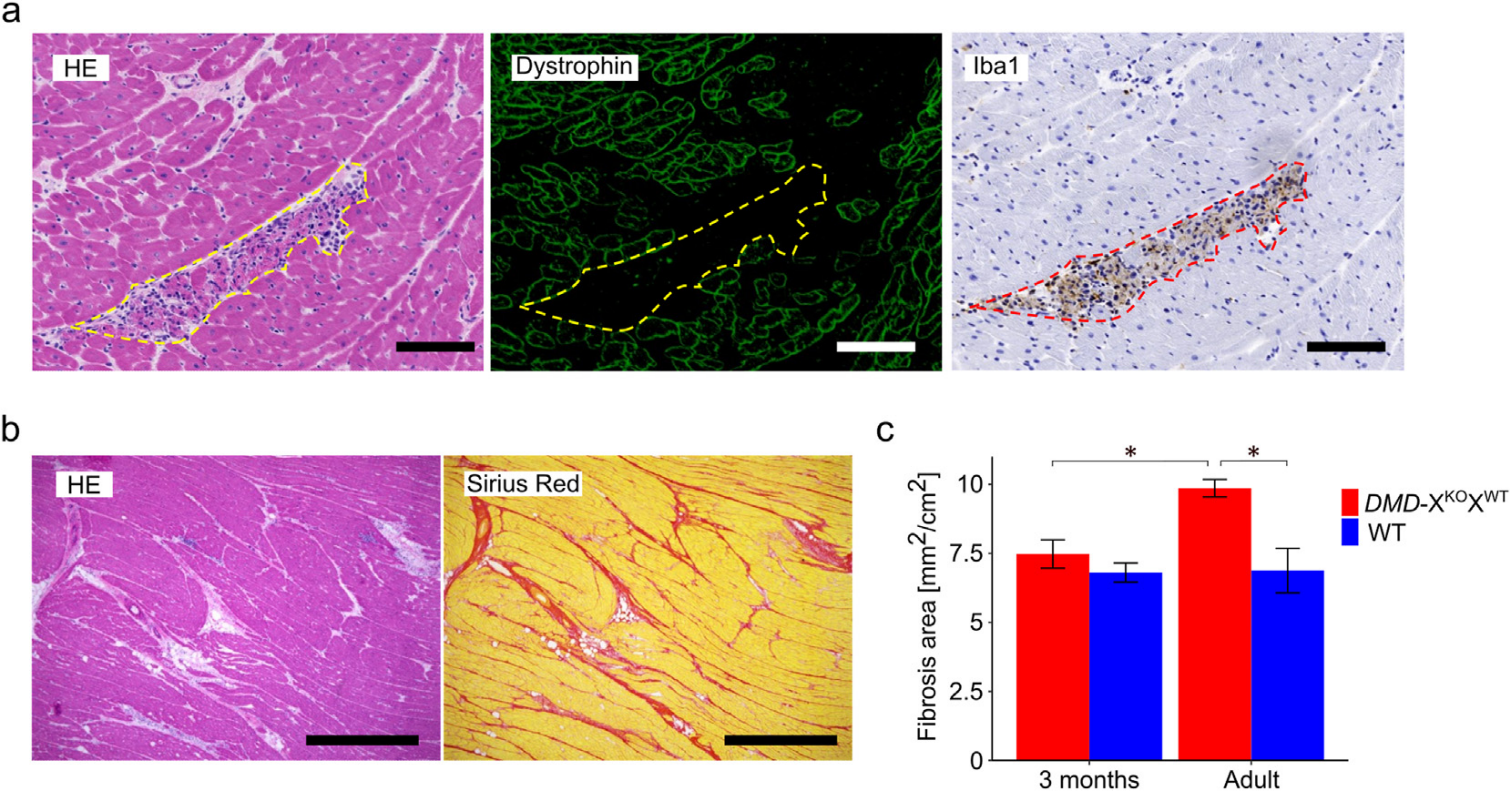
Histopathological analysis of the myocardium of asymptomatic *DMD-*X^KO^X^WT^ pigs. a: Phagocytosis in the myocardium of 14-month-old *DMD-*X^KO^X^WT^ pigs (areas in dashed lines). HE staining (left panel) and immunohistochemical staining of dystrophin and Iba1 (middle and right panels, respectively). Scale bars = 100 μm. b: HE staining (left panel) and Sirius Red staining (right panel) of the myocardium of 14-month-old *DMD-*X^KO^X^WT^ pigs. Scale bars = 1 mm. c: Quantification of fibrosis in the myocardium of *DMD-*X^KO^X^WT^ pigs. The graph represents the mean ± SEM of the fibrotic area. ∗p < 0.05 vs. WT pigs. Samples of the *DMD-*X^KO^X^WT^ pigs were collected from individuals at 3 months (n = 3) and 14–15 months (n = 4) of age (red bars). WT pigs at 3 months (n = 3) and 6–8 months (n = 3) of age (blue bars) were used as the respective controls. HE: hematoxylin–eosin. WT: wild type.

Histopathological analysis of the myocardial tissue of young *DMD-*X^KO^X^WT^ pigs (1.5–3 months old) revealed no notable pathological changes other than slight fibrosis of muscle fibers. However, the myocardial tissue of older individuals (14–32 months old) exhibited phagocytosis in dystrophin-negative areas (Fig. 3a) and degeneration, including fibrosis (Fig. 3b). The fibrotic area in the myocardial tissue of adult *DMD-*X^KO^X^WT^ pigs was expanded compared with that of young individuals (p < 0.05, Fig. 3c).

## 4 Discussion

Although DMD carriers have long been believed to be asymptomatic [6], recent studies have revealed a sporadic incidence of symptom manifestation, including sudden death, in latent patients with the *DMD*-X^KO^X^WT^ genotype. Manifestation of the DMD phenotype in females possessing heterozygous *DMD* mutations has been correlated with a skewed X chromosome inactivation (XCI) pattern [[29], [30], [31]]. This theory suggests that the X chromosomes containing the normal *DMD* are inactivated at a higher rate than those that carry the mutated gene [30]. In contrast, some reports denied a correlation between symptomatic carriers and skewed XCI [[32], [33], [34]]. Thus, a follow-up study on DMD carrier females could provide new information, but such a study would present difficulties from a practical and ethical perspective.

DMD model animals have been developed earlier in mice and dogs [35,36]. Although the *DMD*-X^KO^X^WT^ females in animal models have been reported to exhibit muscle degeneration [[37], [38], [39], [40]], dissimilar pathological features to humans hinder the utility of these animals as faithful models for DMD carriers [41]. Recent development of disease model pigs, including DMD, demonstrated extrapolability of their phenotypes to the symptoms of human patients [19,42,43]. We therefore generated a cohort of *DMD*-X^KO^X^WT^ female pigs in the present study and investigated their eligibility as a DMD carrier model.

*DMD*-X^KO^X^WT^ female pigs were produced in a previous study by using the somatic cell cloning technique [18]. However, most of the founder *DMD*-X^KO^X^WT^ cloned pigs obtained were prematurely mortal, which hindered the production of a cohort of DMD carrier progeny for the study [18]. The mortality of *DMD*-X^KO^X^WT^ cloned pigs is ascribed to abnormal XCI, which is prone to occur in cloned animals [[44], [45], [46]]. Therefore, we generated DMD carrier females as the next-generation offspring of a *DMD*-X^KO^Y↔X^WT^X^WT^ chimeric boar [22]. The use of cryopreserved *DMD*-X^KO^ sperm assures continuous, highly scalable, and economical production of *DMD*-X^KO^X^WT^ female pigs.

Approximately 70% of DMD carrier women exhibit high serum CK levels regardless of clinical manifestation [47]. *DMD*-X^KO^X^WT^ pigs also exhibited frequent hyperCKemia as one of their phenotypic features. In DMD carriers, the reported onset frequency of musculoskeletal abnormalities that range from light muscular pain to severe astasia [7,[48], [49], [50]] varies widely from 2.5 to 19% [6]. This inconsistency may be ascribed to a lack of unity in defining symptoms as a result of the avoidance of invasive muscle biopsy in clinical settings. In the present study, we revealed pathological abnormalities in the skeletal muscles in one-quarter of the *DMD*-X^KO^X^WT^ pigs examined. These data suggest a frequent incidence of pathological abnormalities in the musculoskeletal tissues of latent DMD carrier women.

Clerk et al. [51] reported mosaic expression of dystrophin in the skeletal muscle of asymptomatic DMD carrier infants. In contrast, adult DMD carriers exhibited uniform dystrophin staining in skeletal muscle tissues [51]. These phenomena were explained by the regeneration of myofibers by the activation of dystrophin-positive satellite cells and compensatory dystrophin production by nearby dystrophin-positive myonuclei [52]. A similar transition pattern of dystrophin staining was observed in our *DMD*-X^KO^X^WT^ pigs. Sequential symptomatic analysis in *DMD*-X^KO^X^WT^ female pigs has yet to be performed.

Sporadic manifestation of cardiomyopathy, including dilated cardiomyopathy (DCM), is one of the major features of DMD carriers, and its occurrence increases with age [9,10,53]. Severe DCM cases include mortal consequences of heart failure in middle age [9,10]. Dystrophin is known to be expressed in a mosaic fashion among the myocardial myofibers of DMD carrier females [54]. Because of the lack of regenerative capacity in myocardial fibers [55], the mosaic expression pattern of dystrophin in myocardial tissue is considered to be constant throughout the life of DMD carriers. Necrosis and fibrosis of dystrophin-negative cardiomyocytes are likely to be involved in cardiomyopathy in DMD carriers, similar to male DMD patients [56].

Stirm et al. [18] reported that 15% (3/20) of the *DMD*-X^KO^X^WT^ pigs of which were descended from DMD carrier sows (F1 and F2) exhibited myocardial lesions at 6 months old. In addition, the authors observed severe myocardial abnormalities in the *DMD*-X^KO^X^WT^ sow, which served 7 years. We also noted histological abnormalities in the myocardial tissue in all of the asymptomatic *DMD*-X^KO^X^WT^ pigs (6/6) that had grown up without apparent health problems for 14–32 months after birth. Together, these data may suggest a higher risk of myocardial abnormalities in DMD carrier women than previously thought. The increased workload on the heart of DMD carriers necessary to maintain skeletal muscle may induce cardiomyopathy [57]. The frequent occurrence of histological alterations in the myocardial tissue of *DMD*-X^KO^X^WT^ pigs may underlie the risk of cardiac incompetency in DMD carriers.

## 5 Conclusions

In conclusion, *DMD*-X^KO^X^WT^ pigs exhibit various characteristics similar to DMD carrier patients. Those characteristics included asymptomatic hyperCKemia, dystrophin expression patterns in the skeletal and cardiac muscles, histopathological features of skeletal muscle degeneration, myocardial lesions in adulthood, and sporadic death. Thus, *DMD*-X^KO^X^WT^ pigs could facilitate long-term follow-up studies with invasive interventions, thereby increasing our understanding of the pathogenic mechanisms in asymptomatic carriers and eventually developing biomarkers for stratification of DMD carriers regarding their risk to become symptomatic.

## Declaration of competing interest

H.N. is a founder and shareholder of PorMedTec Co., Ltd. These associations do not alter the authors' adherence to the journal's policies on sharing data and materials. The other authors declare that they have no conflicts of interest.
