# Supplementary material for: Phenotypic features of genetically modified DMD-XKOXWT pigs
Source: Regen Ther. 2023 Sep 20;24:451–8. doi: 10.1016/j.reth.2023.09.010 (PMC10523442; doi:10.1016/j.reth.2023.09.010)
Supplement: Multimedia component 1 [file mmc1.docx]

**Supplementary data**


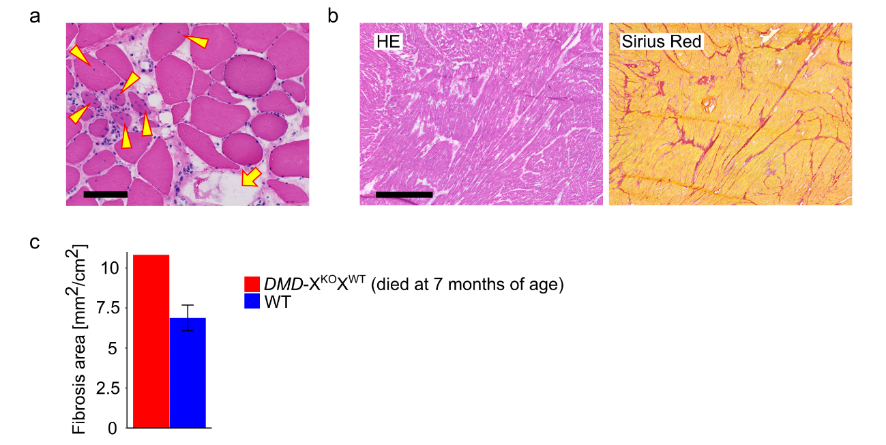


**Supplementary Fig. S1.** Histopathological analysis of the *DMD-*X^KO^X^WT^ pig that died suddenly at 7 months of age. a: HE staining of skeletal muscle lesions. Pathological features of DMD, such as variable myofiber size, centrally nucleated myofibers (arrowheads), and empty spaces representing fat deposition (arrow). Scale bar = 100 µm. b: HE staining (left panel) and Sirius Red staining (right panel) of myocardial tissue demonstrating fibrosis. Scale bar = 1 mm. c: Quantification of myocardial fibrosis. The myocardial fibrotic area of *DMD-*X^KO^X^WT^ pigs (red bar) was compared to that of the WT pigs (6–8 months old, n = 3; blue bar, mean ± SEM). HE: hematoxylin–eosin. WT: wild type.


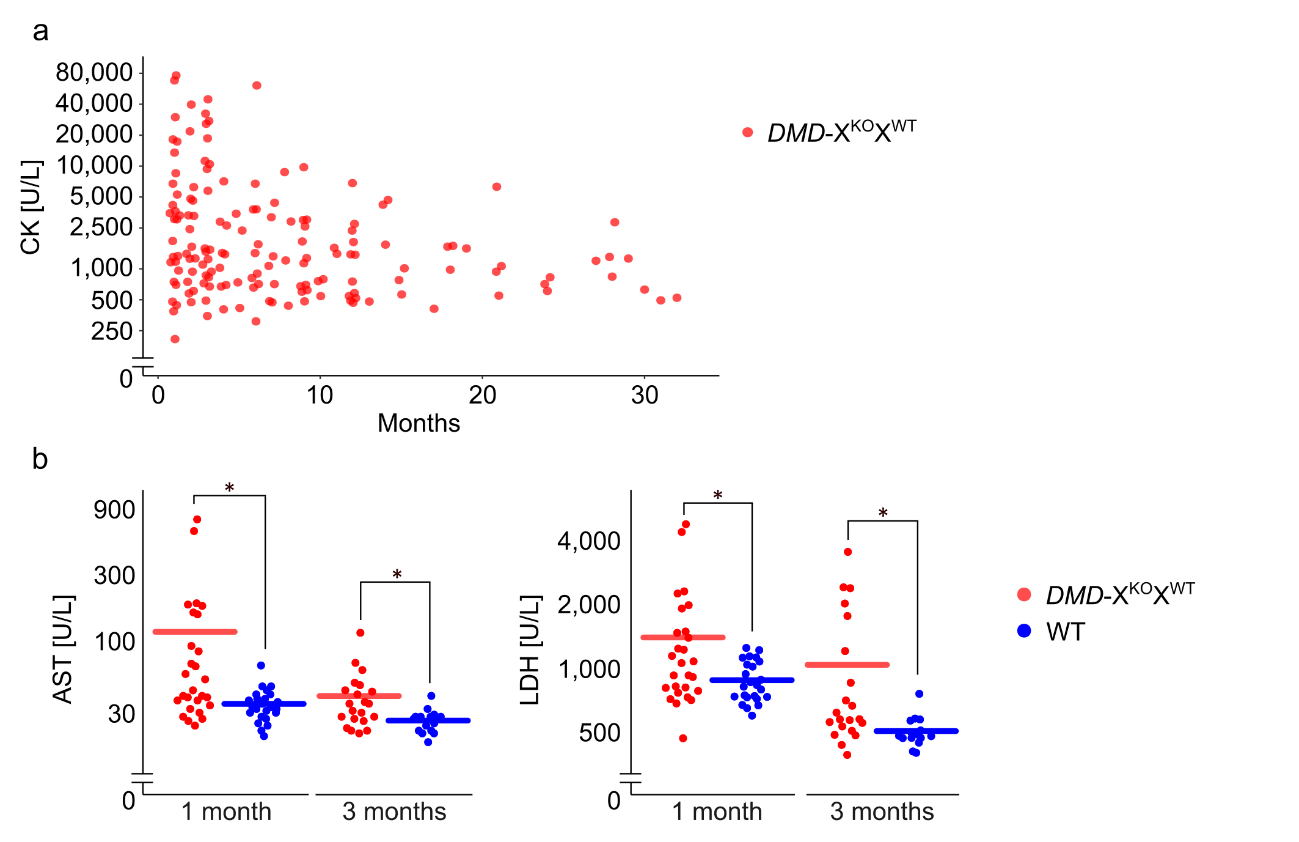


**Supplementary Fig. S2.** a: Dynamics of serum CK levels in *DMD-*X^KO^X^WT^ pigs during the growth period. From a total of 27 *DMD-*X^KO^X^WT^ pigs that survived at least 1 month after birth, blood samples were collected monthly at 1–3 months of age, approximately every 3 months until 12 months of age, and then every 5–6 months at 13–32 months of age. b: Serum AST and LDH levels of young *DMD-*X^KO^X^WT^ female pigs. At young ages, *DMD-*X^KO^X^WT^ pigs (red plots) exhibited significantly higher serum AST levels (1 month old: 118.9 ± 177.9 vs. 35.9 ± 10.0 U/L, 3 months old: 40.9 ± 21.8 vs. 27.2 ± 5.4 U/L) and LDH levels (1 month old: 1,413 ± 1,046 vs. 891 ± 198 U/L, 3 months old: 1,051 ± 871 vs. 513 ± 89 U/L) than those in WT pigs (blue plots). *p < 0.05 vs. WT pigs. Samples were collected from *DMD-*X^KO^X^WT^ pigs at 1 month (n = 27) and 3 months (n = 21) of age. WT pigs at 1 month (n = 24) and 3 months (n = 15) of age were used as the respective controls. CK: creatine kinase, AST: aspartate aminotransferase, LDH: lactate dehydrogenase. WT: wild type.


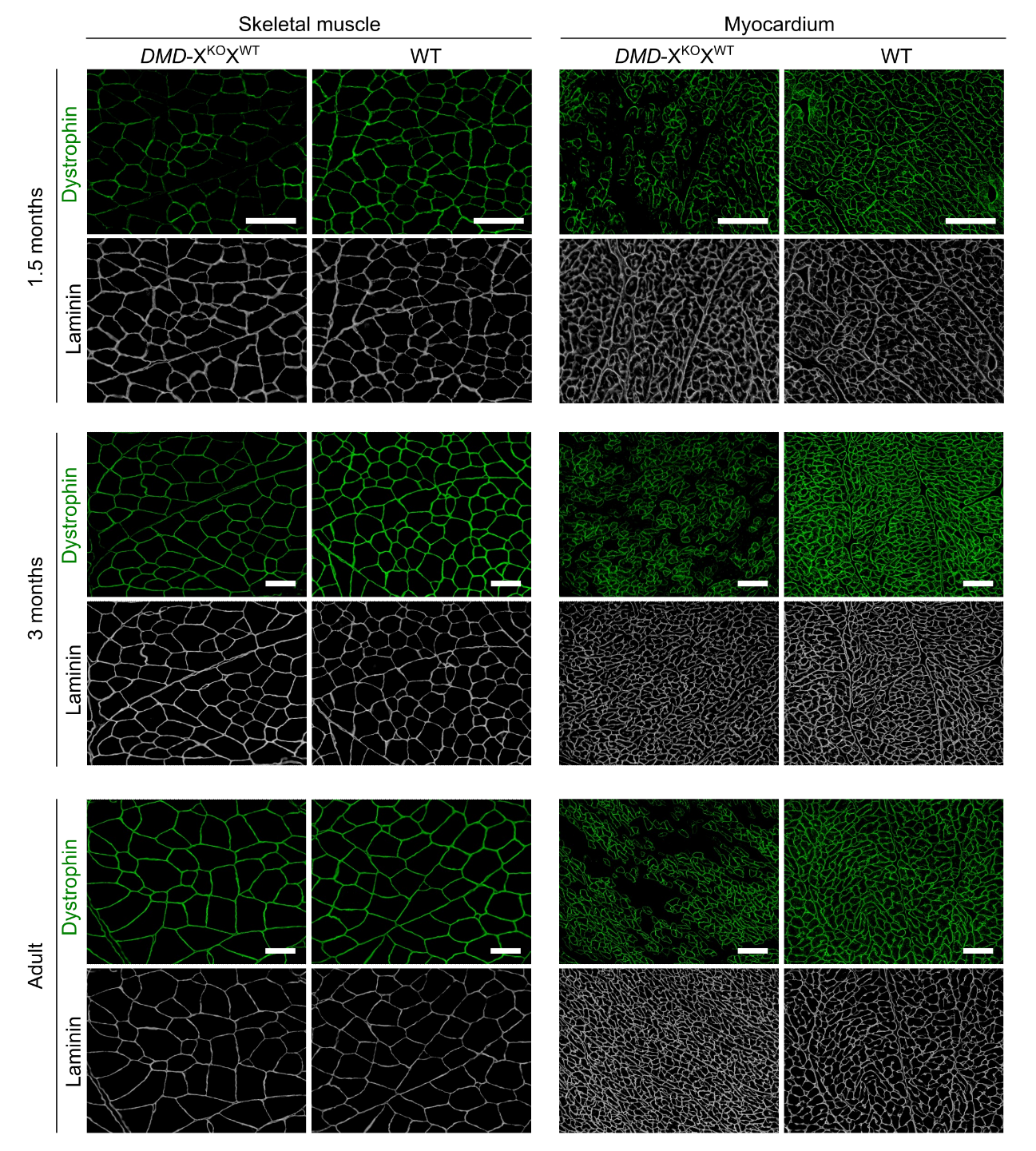


**Supplementary Fig. S3.** Immunostaining of dystrophin and laminin in skeletal muscle and myocardium of *DMD-*X^KO^X^WT^ pigs. The upper and lower images of each age samples exhibit fluorescent immunohistochemical staining of dystrophin and laminin, respectively. Scale bars = 100 µm. WT: wild type.


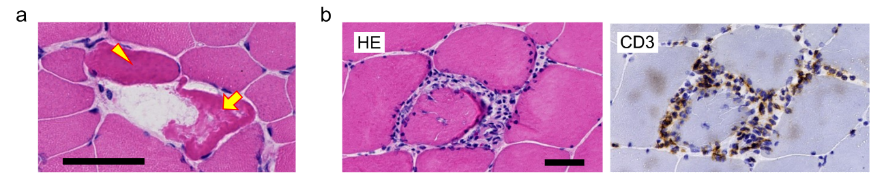


**Supplementary Fig. S4.** Degeneration of muscle fibers in the biceps femoris of asymptomatic *DMD-*X^KO^X^WT^ pigs. a: HE staining of hypercontracted (opaque) fibers (arrowhead) and necrotic fibers (arrow) in young *DMD-*X^KO^X^WT^ pigs (1.5 months old). Scale bar = 50 µm. b: Infiltration of T cells in adult *DMD-*X^KO^X^WT^ pigs (14 months old). HE (left panel) and CD3 immunohistochemical (right panel) staining. Scale bar = 50 µm. HE: hematoxylin–eosin.
